# Supplementary material for: RNA Sequencing of Whole Blood Defines the Signature of High Intensity Exercise at Altitude in Elite Speed Skaters
Source: Genes (Basel). 2022 Mar 24;13(4):574. doi: 10.3390/genes13040574 (PMC9027771; doi:10.3390/genes13040574)
Supplement: Supplementary file 1 [file genes-13-00574-s001.zip › Supplementary Figures S1-S10.pdf]

Supplementary Figures for

RNA sequencing of whole blood defines the signature of high intensity exercise at altitude in elite speed skaters

Andrey S. Glotov[1,2*], Irina E. Zelenkova[3,4], Elena S. Vashukova[2], Anna R. Shuvalova[2], Alexandra D. Zolotareva[2], Dmitrii E. Polev[2], Yury A. Barbitoff[2,5], Oleg S. Glotov[2,6], Andrey M. Sarana[6], Sergey G. Scherbak[6], Mariya A. Rozina[7], Victoria L. Gogotova[7], Alexander V. Predeus[5*]

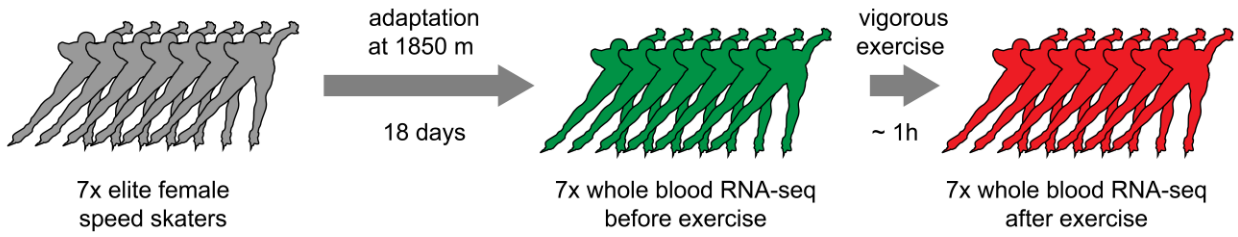

Figure S1. A scheme of the performed experiments. Biochemical blood measurements were taken on days 14, 16, and 18, immediately before and after the exercise bout. Whole blood samples used for RNA sequencing were taken before and after the exercise on day 18.

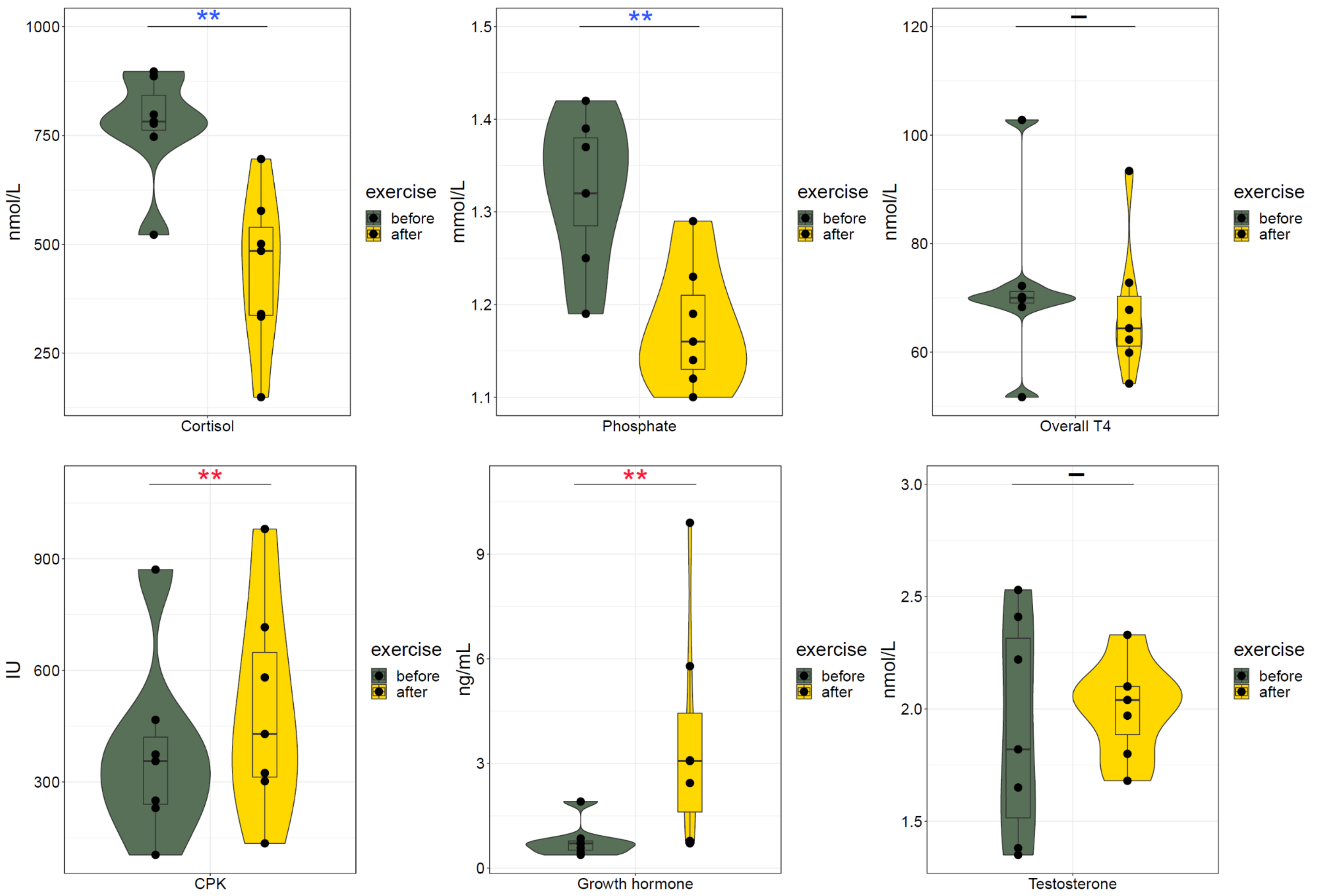

Figure S2. Blood tests taken immediately before and after the exercise bout on day 18. Full table of all performed blood tests is given in Supplementary Table S2. Paired one-sided Wilcoxon test was used to evaluate the significance of each change.

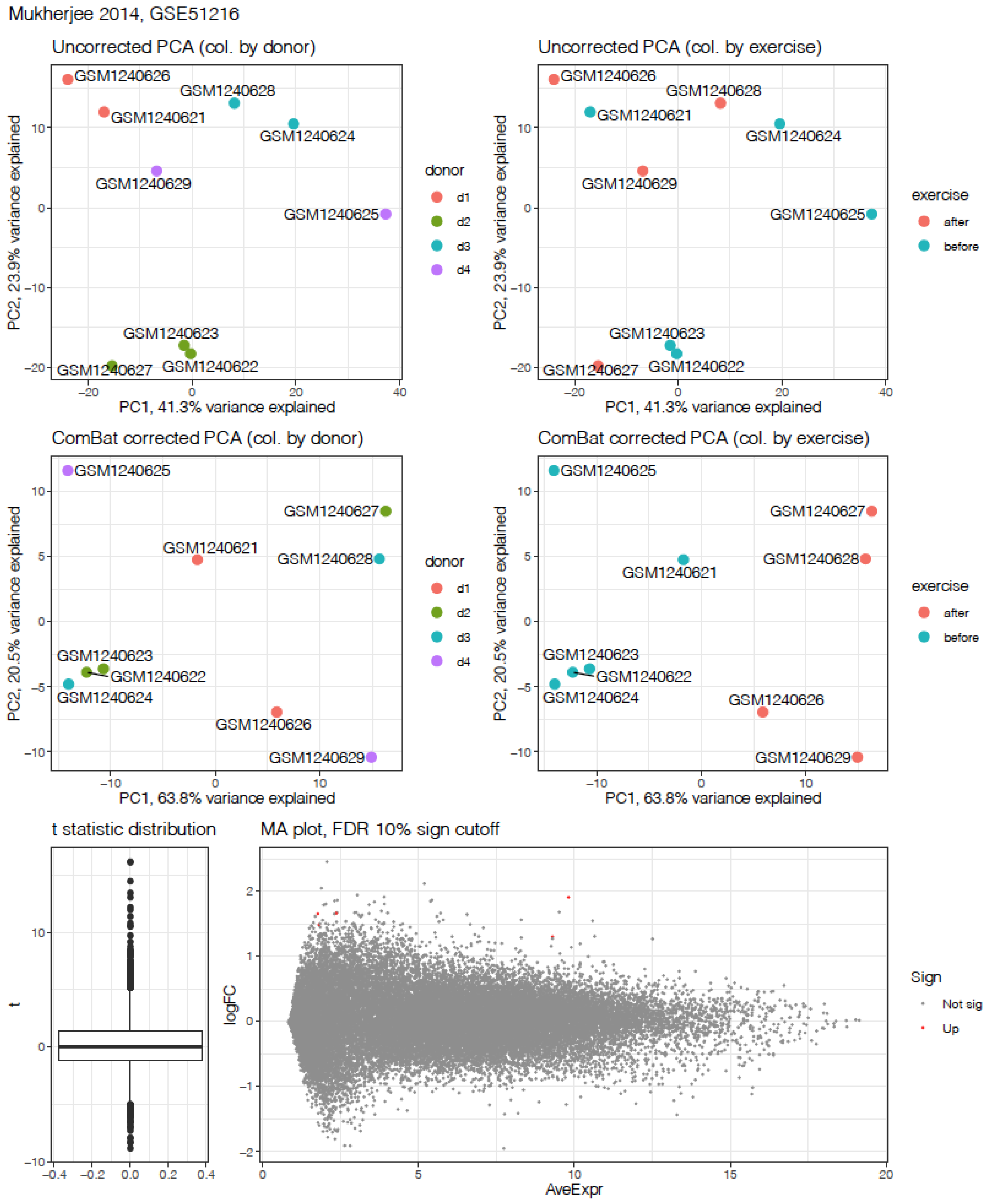

Figure S3. Microarray comparison of 4 adult athletes before and after exercise (Mukherjee 2014).

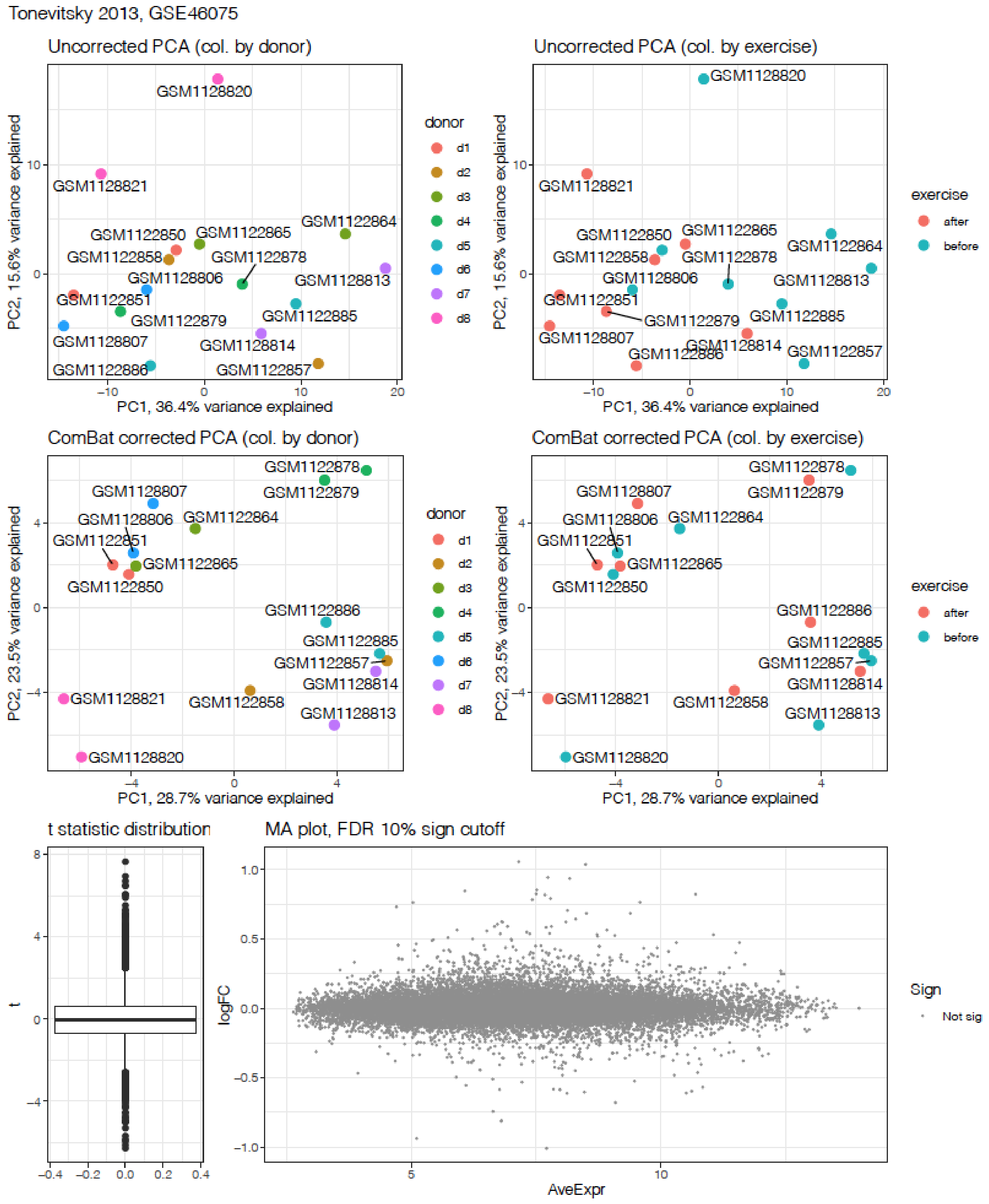

Figure S4. Microarray comparison of 8 adult athletes before and after exercise (Tonevitsky 2013).

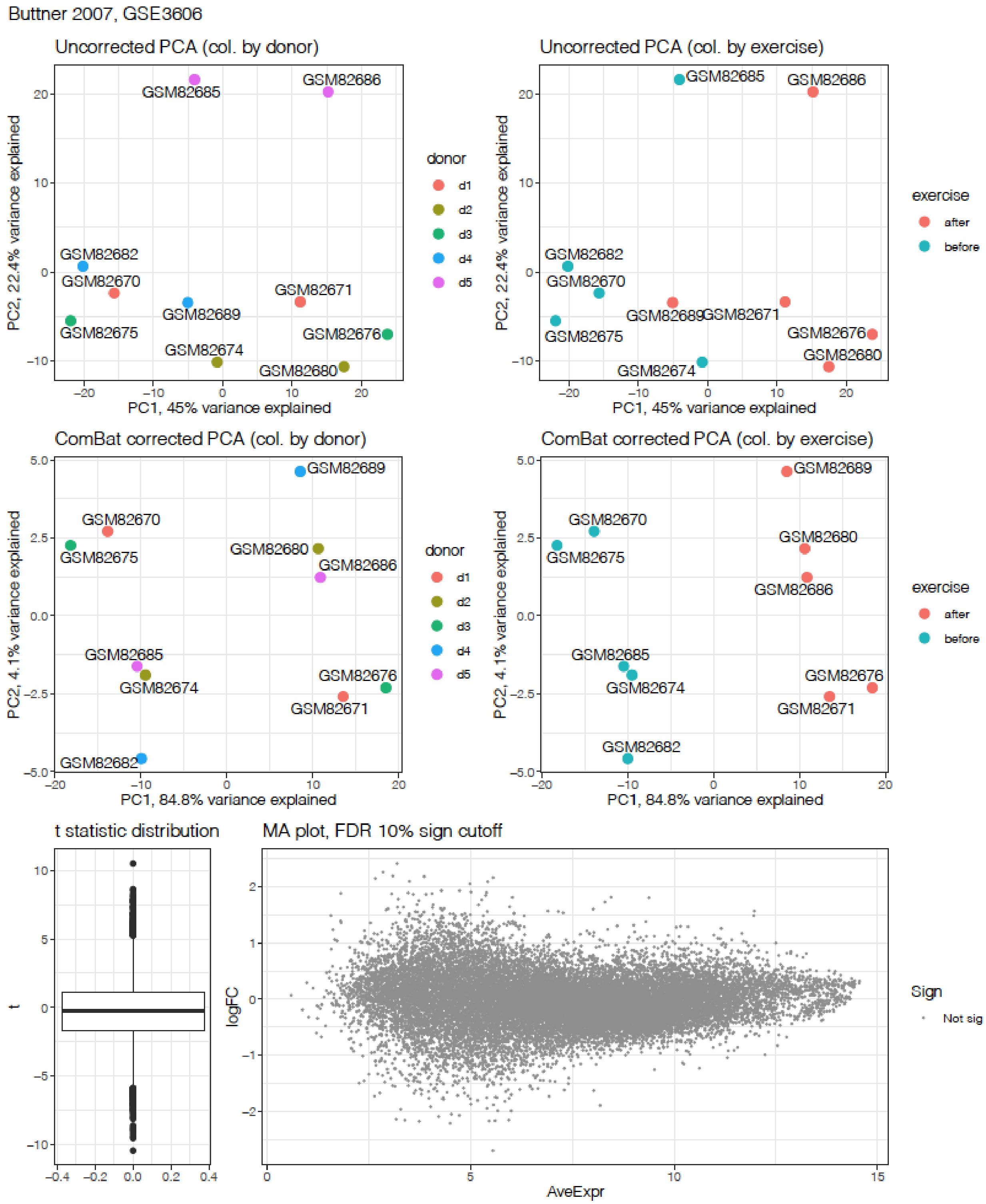

Figure S5. Microarray comparison of 5 adult athletes before and after exercise (Buttner 2007).

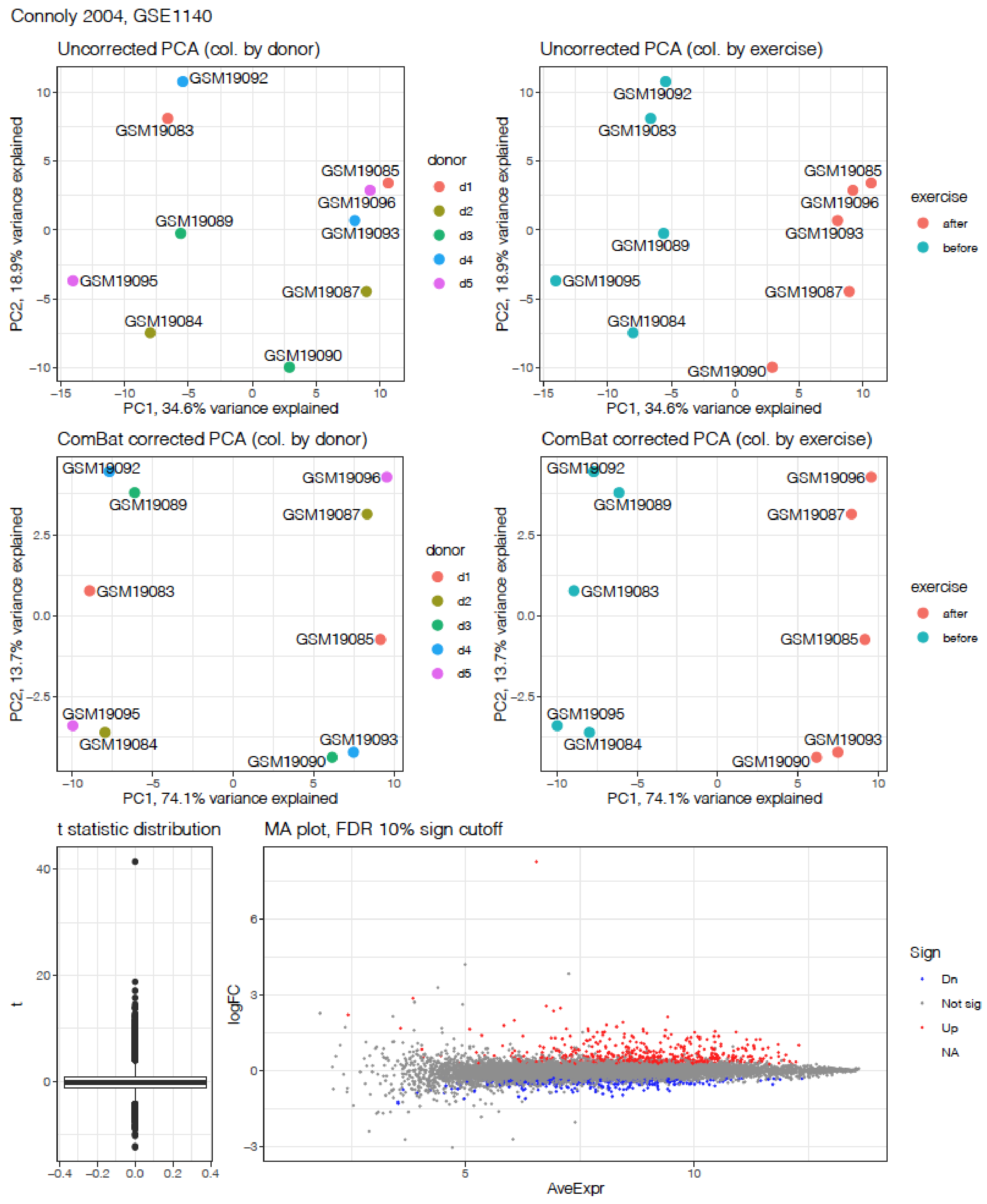

Figure S6. Microarray comparison of 5 adult athletes before and after exercise (Connoly 2004).

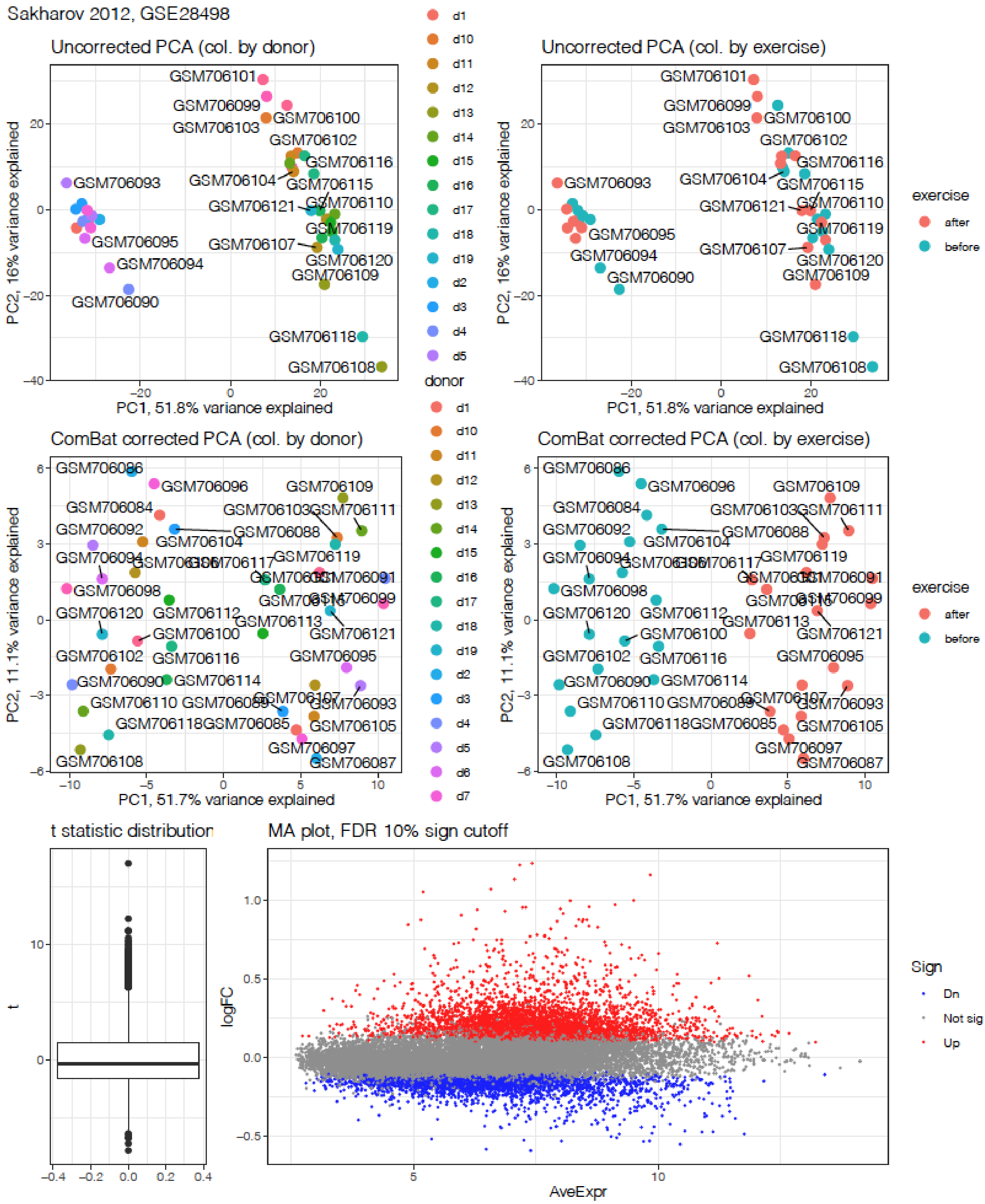

Figure S7. Microarray comparison of 19 adult athletes before and after exercise (Sakharov 2012).

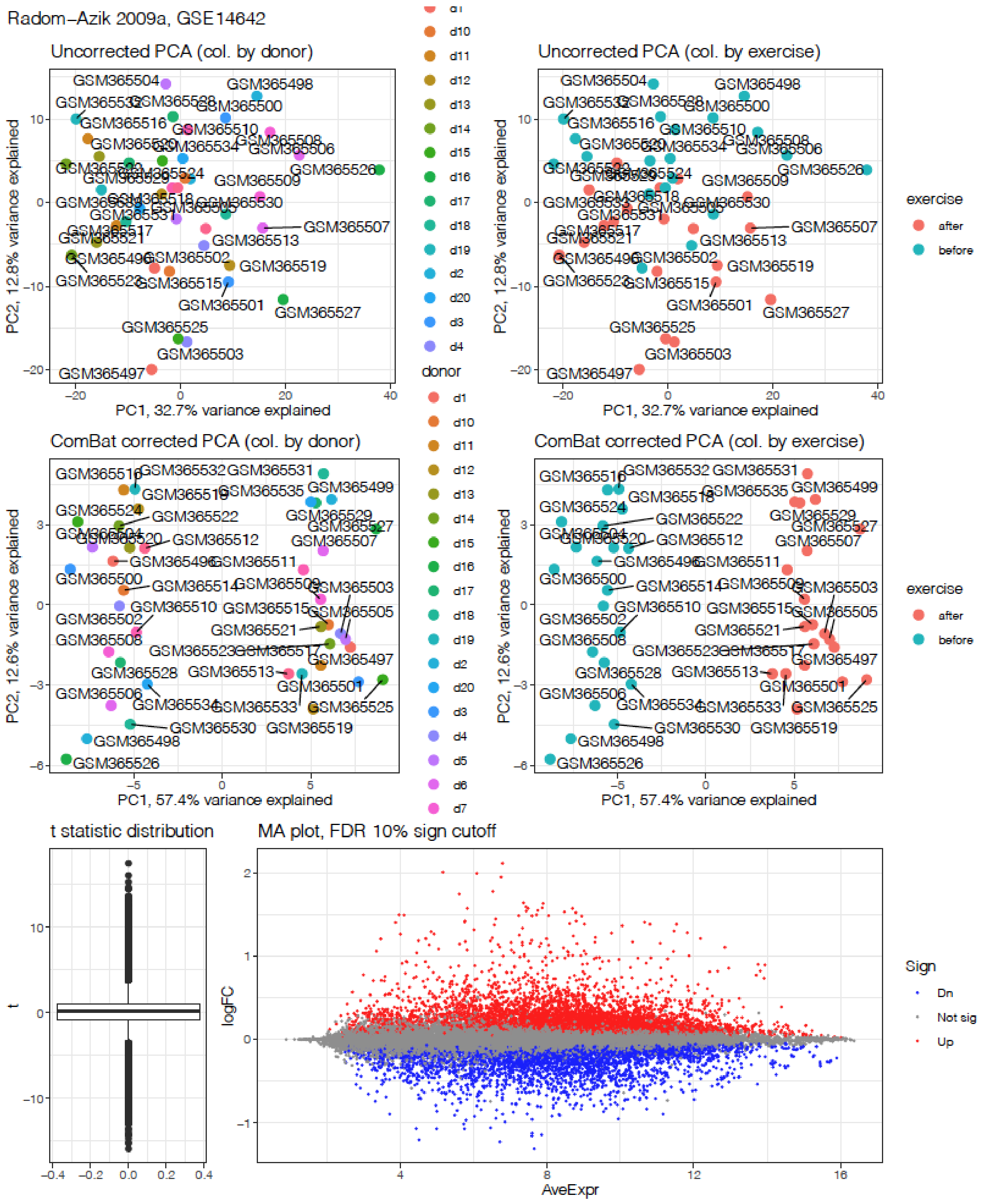

Figure S8. Microarray comparison of 20 teenage athletes before and after exercise (Radom-Azik 2009a).

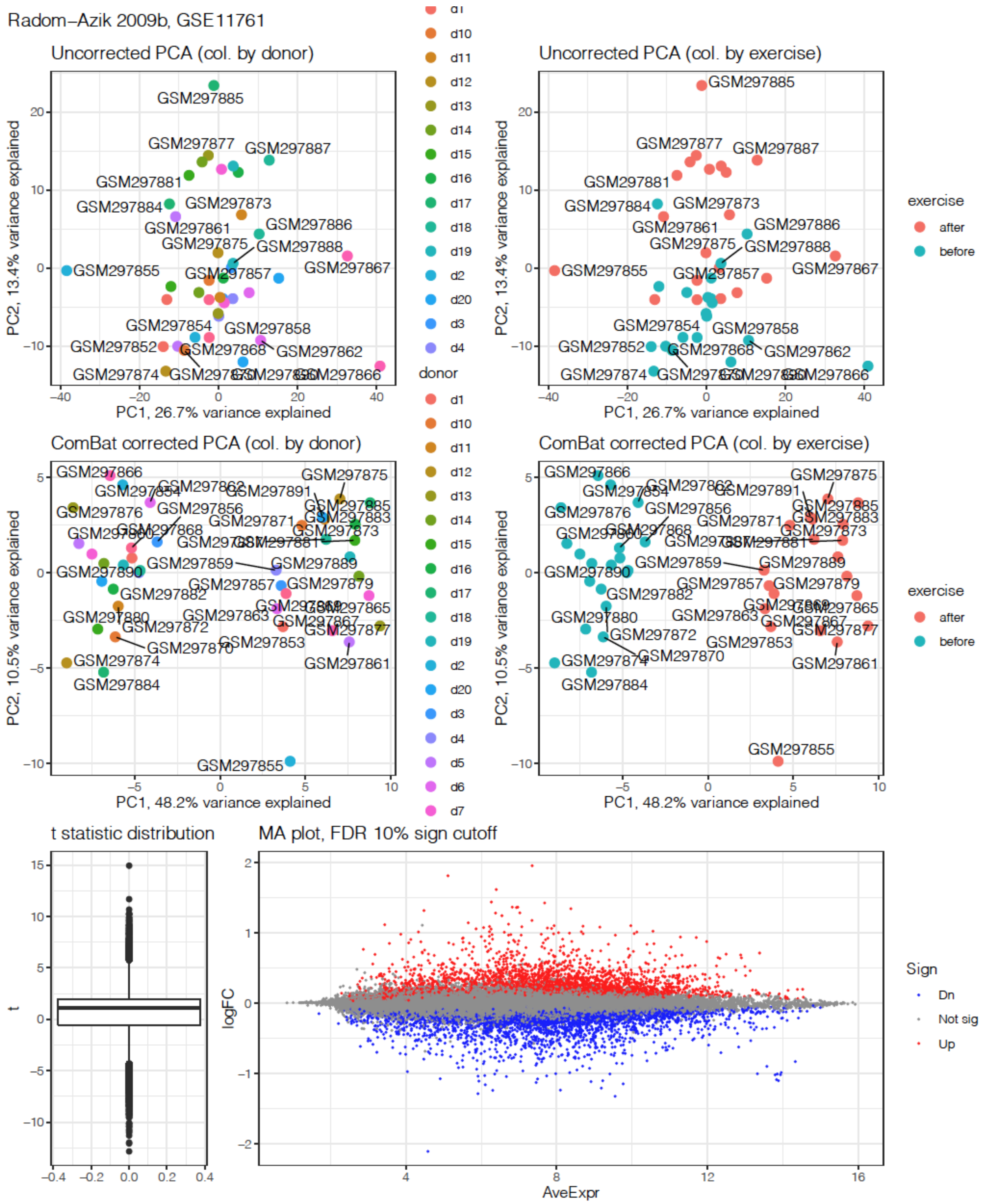

Figure S9. Microarray comparison of 20 teenage athletes before and after exercise (Radom-Azik 2009b).

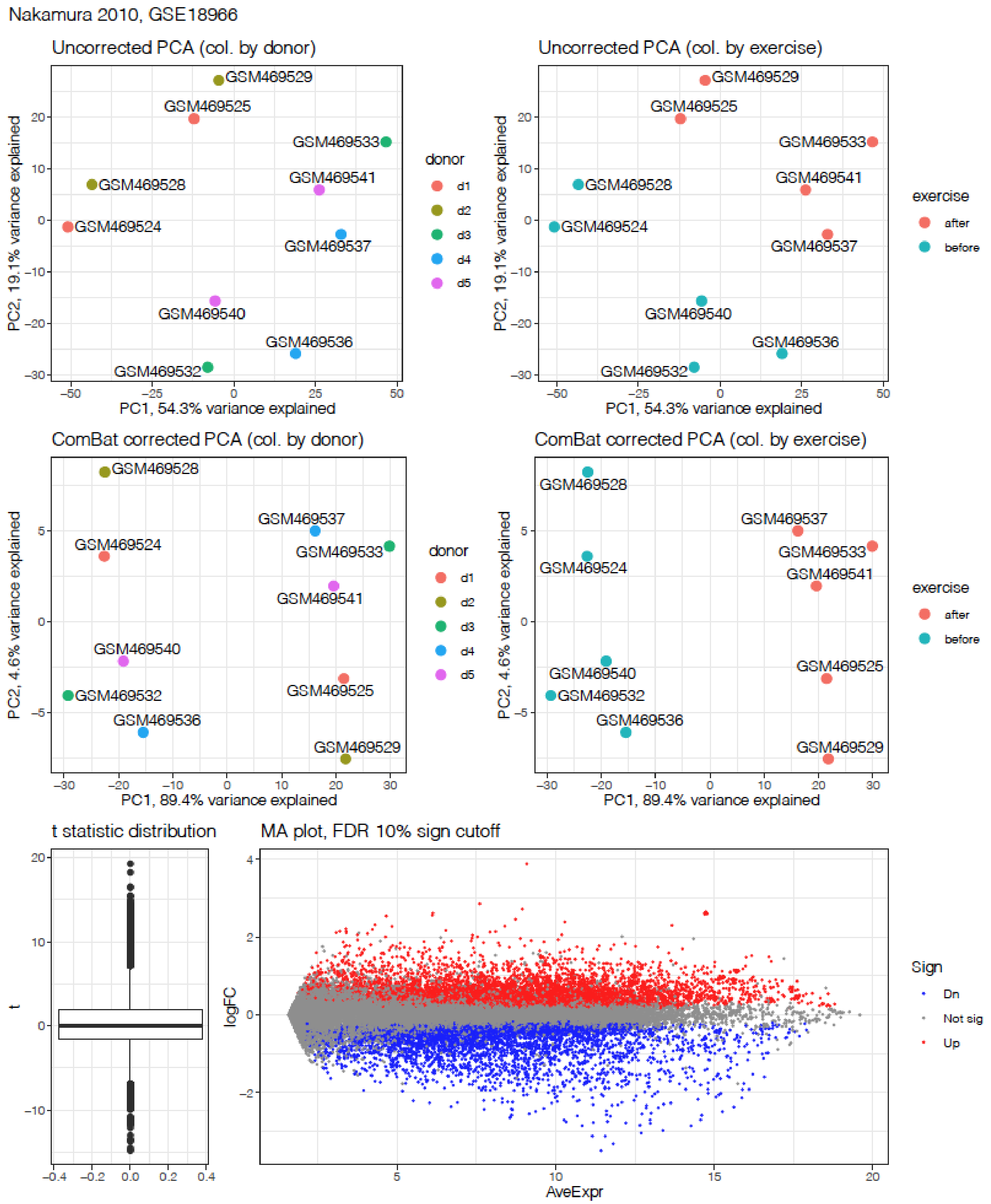

Figure S10. Microarray comparison of 5 adult athletes before and after exercise (Nakamura 2010).
